# Transapical aortic valve implantation: outcome in patients with low arithmetic risk profile

**DOI:** 10.1186/1749-8090-8-S1-O321

**Published:** 2013-09-11

**Authors:** A Unbehaun, M Pasic, T Drews, S Buz, S Dreysse, M Kukucka, A Mladenow, R Hetzer

**Affiliations:** 1 Deutsches Herzzentrum Berlin, Berlin, Germany

## Background

Trancatheter aortic valve implantation (TAVI) has been introduced as an alternative treatment to eliminate aortic stenosis in patients who are at high risk for conventional surgery. Classical risk scores in cardiac surgery do not always reflect the true surgical risk. We analysed outcome and predictors of survival after TAVI in a large single-centre cohort with low arithmetic risk profile.

## Methods

Between 04/2008 and 07/2012, 610 patients (median age 80, range 29-99 years) underwent transapical TAVI. Patients in cardiogenic shock were included (n=33; 5.4%). In accordance with The-Society-of-Thoracic-Surgeons-Predicted-Risk-of-Mortality (STS-PROM-score), the cut-off level between study and control group was arbitrarily set at 10%. The study group of 274 patients (median STS-PROM-score 5.9%, interquartile range [IQR] 4.4%-7.6%) was compared to the control group of 336 patients (median STS-PROM-score 18.0%, IQR 13.5%-26.1%).

## Results

Thirty-day mortality rate in patients with STS-PROM-score<10% was 3.3%. Survival in the study group was significantly better (p<0.001) with 1-, 2-, 3-, and 4-year survival rate of 89%±2%, 83%±3%, 76%±5%, and 72%±6%, respectively. Survival in a sub-group of 89 patients with STS-PROM-score<5% was 95%±3% and 91%±5% at 1 and 3 years; p=0.002. The strongest predictors of mortality in multivariate Cox-regression-analysis were advanced age (p=0.003, hazard ratio [HR] 1.08, 95%-confidence-interval [CI] 1.03-1.14), higher NYHA class (p=0.027, HR 2.54, CI 1.12-5.81), and longer procedural time (p=0.001, HR 1.01, CI 1.01-1.01).

## Conclusions

Regardless of their true surgical risk, patients with low arithmetic risk profile have much better survival after TAVI. This observation is a first prerequisite to broadening the indication for TAVI to patients with low risk for conventional surgery.

